# Preparing for measurement-based care in child and adolescent mental health: a mixed-methods pre-implementation assessment of barriers, facilitators, and implementation strategies

**DOI:** 10.3389/frhs.2026.1984498

**Published:** 2026-10-22

**Authors:** Sabrina Adam, Sadia Ahmed, Fakhriyya Aghabayli, Monica Cepoiu-Martin, Erin McCabe, Jennifer Zwicker, Paul D. Arnold, Gina Dimitropoulos, Ryan Clements, Maria J. Santana

**Affiliations:** 1 Department of Community Health Sciences, Cumming School of Medicine, University of Calgary, Calgary, AB, Canada; 2 Alberta Strategy for Patient-Oriented Research Support Unit, Calgary, AB, Canada; 3 Department of Physical Therapy, Faculty of Rehabilitation Medicine, University of Alberta, Edmonton, AB, Canada; 4 Alberta Children's Hospital Research Institute, Calgary, AB, Canada; 5 School of Public Policy, University of Calgary, Calgary, AB, Canada; 6 Faculty of Kinesiology, University of Calgary, Calgary, AB, Canada; 7 Department of Psychiatry, Cumming School of Medicine, University of Calgary, Calgary, AB, Canada; 8 Mathison Centre for Mental Health Research and Education, Cumming School of Medicine, University of Calgary, Calgary, AB, Canada; 9 Cumming School of Medicine, University of Calgary, Calgary, AB, Canada; 10 Alberta Health Services, Calgary, AB, Canada

**Keywords:** child and adolescent mental health, COM-B, consolidated framework for implementation research, implementation, measurement based care, patient reported outcome measures, pre-implementation

## Abstract

**Background:**

Measurement-Based Care (MBC) uses patient-reported outcome measures (PROMs) to routinely monitor patient symptoms and functioning over time and support clinical decision-making. The implementation of MBC in child and adolescent mental health care in Alberta remains sparse, with challenges arising particularly when workflows, staffing, and available resources differ across clinics. This study assessed readiness for MBC implementation at a child and adolescent mental health program and explored the barriers and facilitators that would need to be considered during implementation planning.

**Methods:**

A mixed-method pre-implementation assessment was conducted at the Children's Day Treatment Program (CDTP) within the Alberta Children's Hospital. Staff at this clinic completed a readiness survey assessing their attitudes towards the implementation of MBC at the CDTP clinic, followed by a semi-structured interview conducted by the research team to explore potential barriers and facilitators to implementation. Data from the survey were summarized using descriptive statistics and data from the interviews were transcribed and coded into the Consolidated Framework for Implementation Research (CFIR) 2.0 framework to assess barriers and facilitators. After this assessment, barriers were then mapped into the Capability, Opportunity, Motivation-Behaviour (COM-B) framework to inform implementation strategies.

**Results:**

Thirteen CDTP clinic staff participated in the readiness assessment; ten staff completed the survey and 13 participated in the interview. Staff generally viewed MBC positively and felt that it aligned with the clinic's emphasis on evidence-based care and monitoring patient progress. However, staff also identified potential barriers to implementation, including integrating MBC into the clinic's current workflow, clarifying staff responsibilities for the administration of measures, and providing training on the specific measures being used. These findings were used to inform implementation strategies that address staff concerns and support the integration of MBC into the existing CDTP workflow.

**Conclusion:**

The readiness assessment showed that while staff were supportive of MBC, there were also practical issues that needed to be addressed before implementation. Involving clinic staff early helped the research team understand the barriers specific to CDTP and make changes to the implementation strategies based on the clinic's existing workflow and resources. Future stages of the INSIGHT project will assess how well these strategies work in practice and how they support the implementation of MBC.

## Introduction

Mental health conditions represent a substantial burden among children and adolescents, with approximately one in seven youth aged 10–19 years experiencing a mental disorder globally (1). Depression, anxiety, and behavioural disorders are among the leading causes of illness and disability during adolescence, and untreated mental health conditions can have lasting consequences for health and functioning into adulthood (1). Given the prevalence and potential long-term impact of mental health concerns during this developmental period, approaches that support the ongoing assessment of symptoms and functioning are particularly important within child and adolescent mental health services to understand how children and adolescents are progressing throughout care (2).

Measurement-Based Care (MBC) is an evidence-based, patient-centred approach that uses standardized patient-reported outcome measures (PROMs) to routinely monitor symptoms, functioning, and treatment progress throughout care (3, 4). PROMs are standardized questionnaires completed by patients and/or caregivers that capture health outcomes directly from the perspective of the individual completing them. In this way, routine collection and discussion of PROMs can bring the perspectives of youth and caregivers into clinical care, making outcomes that matter to patients and families visible alongside clinical judgement. Reviewing these perspectives over time can also support a shared understanding of treatment progress and provide opportunities for youth, caregivers, and clinicians to discuss whether care is addressing the concerns and goals that are most important to them. By collecting and reviewing PROM data over time, MBC provides clinicians with objective information to complement clinical judgement and guide treatment adjustments based on patients’ changing needs (3). The growing interest in integrating PROMs into electronic health records is reflected in a recent systematic review protocol examining how this integration may influence clinical care and patient outcomes (5). These findings show the potential of MBC to improve the quality and delivery of mental health care when PROM data are routinely integrated into clinical practice. However, realizing these benefits depends on the successful integration of MBC into routine clinical practice, which remains challenging across mental health care settings (4, 6).

These challenges are particularly relevant within Child and Adolescent Mental Health Services (CAMHS), where the implementation of MBC is still limited (6). In Alberta, this implementation gap is also clear at the provincial level. A 2023 report from the School of Public Policy found that Alberta lacks a unified approach to measuring and monitoring child and youth mental health outcomes across programs (7). Instead, organizations across the province reported using different outcome measures, or none, making it difficult to compare outcomes across programs and evaluate service effectiveness. However, efforts to integrate MBC into child and adolescent mental health care have recently emerged in Alberta. For example, The Summit: Marian & Jim Sinneave Centre for Youth Resilience in Calgary has incorporated PROMs into its development as a learning health system (8).

More recently, Bajgain et al. (9) described a protocol for implementing MBC within a specialized child and adolescent OCD program in Calgary, incorporating organizational readiness assessment and CFIR 2.0-informed assessment of contextual factors. Together, these initiatives highlight growing efforts to implement MBC within Alberta's child and adolescent mental health services, while also demonstrating the importance of considering the specific clinical and organizational context of individual programs.

The objective of this study was to assess the readiness for MBC of a child and adolescent mental health program in Calgary, identify barriers and facilitators to implementation, and to translate these findings into context specific strategies to support the implementation of MBC into routine clinical workflow. This paper reports findings from the pre-implementation phase of The Implementing and Evaluating an Adolescent and Child Measurement-Based Care Program in Alberta's Mental Health Care System (INSIGHT) study within one site, Children's Day Treatment Program (CDTP) at the Alberta Children's Hospital (ACH). The findings were then used to inform implementation planning and identify strategies to support the integration of MBC into the clinics existing workflow.

## Methods

This study employed a mixed-methods pre-implementation readiness assessment guided by the Consolidated Framework for Implementation Research 2.0 (CFIR 2.0) (10) and the Capability, Opportunity, Motivation–Behaviour (COM-B) framework (11). The CFIR 2.0 was used to identify barriers and facilitators to implementation reported by CDTP staff, while COM-B was used to map these findings to the behavioural determinants of implementation.

### Ethics statement

This study received ethics approval from the University of Calgary Conjoint Health Research Ethics Board (REB25-1050). All participants provided informed consent before participating in the readiness assessment survey and semi-structured interviews. Participants were informed of the purpose of the study, what participation involved, and the voluntary nature of their participation. All interviews were audio-recorded using Zoom with participants’ permission, transcribed verbatim, and de-identified prior to analysis.

### Study design and setting

This study is embedded within a larger multi-step research project using a mixed-methods approach. The INSIGHT project consists of four phases: pre-implementation, implementation, evaluation, and post-implementation (see Figure 1 for a project overview). The purpose of this work focuses on the pre-implementation phase to identify factors that could influence successful implementation as well as potential barriers requiring additional support. Understanding these factors was important for informing implementation strategies. The implementation phase focuses on the delivery of MBC within participating clinics and the evaluation of implementation outcomes. Data sources will include youth and caregiver patient-reported outcome measures (PROMs), clinical staff surveys, electronic health record (EHR) data, patient experience surveys, administrative health data, and in-clinic observations. These data will be used to evaluate implementation outcomes and examine the impact of MBC on clinical care. The evaluation phase focuses on understanding determinants of implementation success, followed by the post-implementation phase, which focuses on the sustainability of MBC following implementation. This last phase includes an assessment of long-term sustainability and the factors that contribute to successful integration of MBC into routine clinical practice

**Figure 1:**
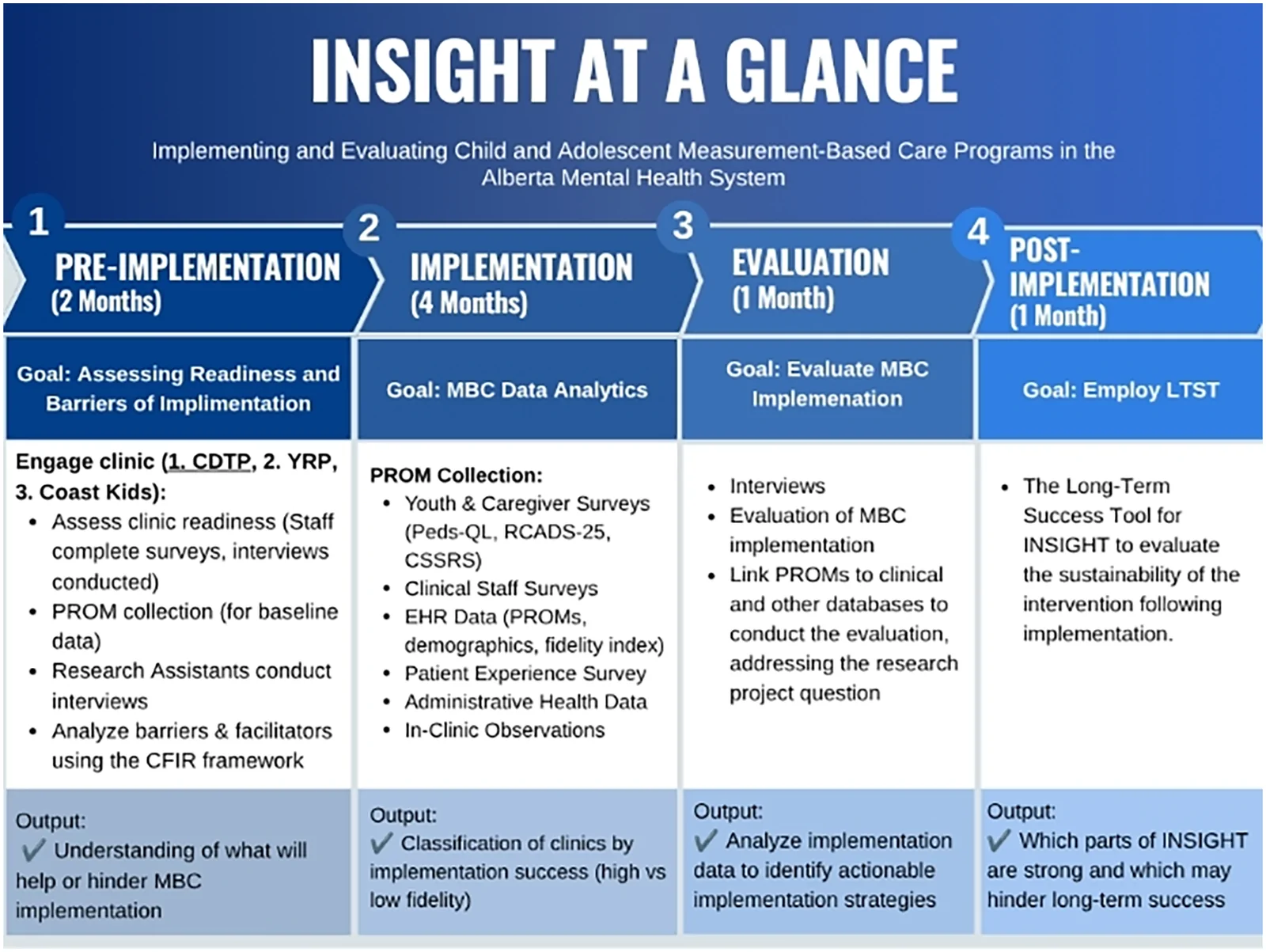
An overview of the INSIGHT project's phases.

### Context: overview of children's day treatment program

CDTP was the first of three clinics selected for MBC implementation as part of a broader initiative within the Child and Adolescent Addictions, Mental Health, and Psychiatry Program (CAAMHPP). The program serves diverse child and adolescent populations, with differences in the age ranges and primary diagnoses represented across clinics. The primary diagnoses varied by clinic and included obsessive-compulsive disorder (OCD), depression, anxiety, attention-deficit/hyperactivity disorder (ADHD), autism, and behavioural problems.

CDTP is a school-based day treatment program that provides assessment and treatment for children, adolescents, experiencing mental health concerns and their families. The clinical team consists of psychologists, psychiatrists, nurses, family counsellors, occupational therapists, psychometrists, and other health professionals. The program also includes two Calgary Board of Education teachers who work with the clinical team to support students’ educational and therapeutic needs.

The research team partnered with Recovery Alberta and Power BI team to support the development of the REDCap data collection system and Power BI data visualizations. Recovery Alberta provided organizational support for implementation.

### Participants and data collection

To assess MBC implementation readiness, multiple data collection methods were used, including staff surveys and semi-structured interviews. Participants were purposively selected by the program manager based on their roles within the clinic and their anticipated involvement in the delivery and/or support of MBC to take part in an initial meeting before pre-implementation with the research team. During this meeting, staff members who would be directly involved in implementing MBC in routine clinical practice were invited to participate in the pre-implementation process, including the survey and interviews. Participation was voluntary, and staff were given the option to decline participation. Staff who consented to participate notified their manager, who subsequently provided the research team with a list of consenting staff members to contact. The research team was therefore not informed of which staff members had declined participation.

#### Survey

Thirteen of the 29 current non-casual staff members were invited to complete an anonymous structured survey, and 10 completed it. The survey was distributed via staff email by a member of the research team and administered through the University of Calgary Qualtrics platform (Table 1). Participants were informed that their survey responses would remain anonymous and could not be linked to their individual identities. The survey collected demographic information, including professional role, and assessed several domains related to MBC implementation. These included perceptions of treatment monitoring and feedback, beliefs regarding the impact of MBC on patients and caregivers, perceived utility of standardized outcome measures, barriers to using standardized assessments in clinical practice, and self-reported confidence in completing key MBC-related tasks. Questions from the survey were drawn from three validated measures: the Monitoring and Feedback Attitudes Scale (MFA), the Attitudes Toward Standardized Assessment Scales–Monitoring and Feedback (ASA-MF), and the MBC Self-Efficacy Scale (12). Responses were collected using 5-point Likert scales ranging from strongly agree to strongly disagree or from not at all confident to very confident, depending on the survey section. Survey findings were used to identify potential facilitators and barriers to implementation and to complement findings from the readiness assessment interviews. Survey responses were summarized using descriptive statistics (frequencies, percentages) in tables and figures.

**Table 1:** Staff survey results assessing perceptions, attitudes, barriers, facilitators and readiness for MBC implementation at the CDTP Clinic (*n* = 10)

Q #	Statement	Disagree *n* (%)	Neutral *n* (%)	Agree *n* (%)
Current MBC Training and Practice. Staff self-reported training, knowledge, and the day-to-day use of MBC, including whether the scores are actively used and discussed with patients (*n* = 10).				
1	I am trained in the use of MBC and how to use scores for clinical decisions	4 (40)	2 (20)	4 (40)
2	I have sufficient knowledge to be able to interpret MBC scores	4 (40)	3 (30)	3 (30)
3	I use MBC at each visit to monitor the course of treatment for patients	7 (70)	3 (30)	0 (0)
4	I discuss MBC scores with my patients	4 (40)	5 (50)	1 (10)
Perceived Value and Benefits of MBC and Feedback. Staff beliefs about the overall importance of MBC and the benefits of providing patients and caregivers with feedback on treatment progress (*n* = 10).				
5	MBC is an important part of treatment	0 (0)	2 (20)	8 (80)
6	MBC is valuable for reflecting on my practice	0 (0)	2 (20)	8 (80)
7	Providing feedback helps increase patient motivation and engagement	0 (0)	5 (50)	5 (50)
8	Providing feedback helps patients/caregivers make informed decisions about their care	0 (0)	2 (20)	8 (80)
9	Providing feedback facilitates collaboration between patients/caregivers and clinical staff	0 (0)	2 (20)	8 (80)
10	Patients/caregivers want their provider to give information about treatment progress	0 (0)	3 (30)	7 (70)
11	Providing feedback can increase patient insight	0 (0)	3 (30)	7 (70)
12	Providing feedback helps keep treatment focused on goals	0 (0)	4 (40)	6 (60)
13	Providing feedback creates an expectation for positive change	0 (0)	3 (30)	7 (70)
14	Providing feedback can lead to better treatment outcomes	0 (0)	5 (50)	5 (50)
Concerns About Providing Feedback to Patients and Caregivers. Staff-reported concerns about potential harms of sharing progress feedback to patients and caregivers (*n* = 10).				
15	Providing feedback would potentially harm the therapeutic alliance	5 (50)	4 (40)	1 (10)
16	Providing negative feedback would lead to patient deterioration	4 (40)	6 (60)	0 (0)
17	Providing negative feedback would decrease patient motivation or engagement	2 (20)	6 (60)	2 (20)
18	Providing negative feedback would make patients think their clinician is incompetent	4 (40)	4 (40)	2 (20)
Perceived Utility and Validity of Using Standardized Measures. Staff beliefs about whether standardized measures provide meaningful clinical information.				
19*	Standardized measures don't tell me anything I can't learn from talking to patients	4 (44)	4 (44)	1 (11)
20	Standardized measures provide more useful information than informal interviews	2 (20)	7 (70)	1 (10)
21	Standardized measures don't capture what's really going on with patients	3 (30)	6 (60)	1 (10)
22	Standardized measures gather information that may not otherwise come up in session	0 (0)	3 (30)	7 (70)
23	Standardized measures are not able to detect meaningful changes as they occur	3 (30)	5 (50)	2 (20)
24	Standardized measures don't measure outcomes most important to patients	2 (20)	8 (80)	0 (0)
25	Standardized measures help gather objective information about whether treatment is working	0 (0)	5 (50)	5 (50)
Practical Utility of Standardized Measures in Clinical Workflow. Staff perceptions of how well standardized measures integrate into session-level practice (*n* = 10).				
26	Standardized measures interfere with establishing rapport during a session	6 (60)	4 (40)	0 (0)
27	Standardized measures help identify when treatment is not going well	0 (0)	6 (60)	4 (40)
28	Standardized measures can help plan for sessions	0 (0)	5 (50)	5 (50)
29	Standardized measures help identify when to change the overall treatment plan	1 (10)	4 (40)	5 (50)
30	Standardized measures can efficiently gather information	2 (20)	3 (30)	5 (50)
31	Standardized measures provide helpful information about when to terminate treatment	0 (0)	6 (60)	4 (40)
Perceived Barriers to MBC Use. Staff-reported downsides and practical barriers to routine MBC use, including limitations of the tool, time, and work burden.				
32	Using clinical judgment to monitor progress is superior to using standardized measures	2 (20)	5 (50)	3 (30)
33	Clinical problems are too complex to be captured by a standardized progress measure	2 (20)	6 (60)	2 (20)
34	The information from standardized measures isn't worth the time spent	6 (60)	4 (40)	0 (0)
35	Completing standardized measures is too much of a burden for clients	4 (40)	4 (40)	2 (20)
36*	I do not have time to administer standardized measures on a frequent basis	1 (11)	6 (67)	2 (22)
Clinic Readiness for MBC Implementation. Staff perceptions of organizational readiness, such as fit with clinic, leadership support, staffing capacity, and access to technology and training resources (*n* = 10).				
37	MBC fits well with our clinic's values and approach to care	0 (0)	3 (30)	7 (70)
38	MBC seems manageable to use within daily clinical workflow	2 (20)	3 (30)	5 (50)
39	Having opportunity to adjust MBC process during early implementation would be helpful	0 (0)	3 (30)	7 (70)
40	Implementing MBC is a high priority for our clinic	0 (0)	7 (70)	3 (30)
41	Leadership clearly communicates that MBC is an important initiative	0 (0)	3 (30)	7 (70)
42	We have enough staff to successfully implement MBC	0 (0)	5 (50)	5 (50)
43*	My current workload allows me to participate in MBC implementation	1 (11)	5 (56)	3 (33)
44	Leaders are accessible and responsive and help remove barriers to implementation	0 (0)	2 (20)	8 (80)
45	We have adequate technology to support MBC	4 (40)	6 (60)	0 (0)
46	We have access to training resources related to MBC	1 (10)	6 (60)	3 (30)
47	Communication about MBC is clear and timely	1 (10)	5 (50)	4 (40)
48	Roles and responsibilities related to MBC are well defined	4 (40)	6 (60)	0 (0)

Staff self-rated Confidence in the Core MBC Tasks						
Q #	Task	Not at All *n* (%)	Slight Extent *n* (%)	Moderate Extent *n* (%)	Great Extent *n* (%)	Very Great Extent *n* (%)
49**	Administer the MBC measure	0 (0)	1 (12)	2 (25)	4 (50)	1 (12)
50*	View the client's MBC score/graph before the session	3 (33)	0 (0)	3 (33)	2 (22)	1 (11)
51	Give feedback about MBC results to client/caregiver	4 (40)	0 (0)	3 (30)	2 (20)	1 (10)
52	Use MBC data to inform treatment planning	2 (20)	2 (20)	1 (10)	4 (40)	1 (10)

Disagree=Strongly Disagree+Disagree; Agree=Agree+Strongly Agree; Neutral=Neither Agree nor Disagree. *n* = 10 for all items unless otherwise indicated.

* *n* = 9; ** *n* = 8, due to missing responses.

#### Semi-structured interviews

Following completion of the staff surveys, between March 17 and April 3, 2026, two researchers conducted readiness assessment interviews with 13 staff members representing a range of professional roles, including 1 Manager, 2 Nurse Clinicians, 2 Registered Nurses, 1 Clinical Supervisor, 1 Family Liaison, 2 Psychologists, 1 Psychometrist, and 3 Family Counsellors. Survey findings were reviewed prior to the interviews and informed areas for further discussion during the readiness assessment process. The survey and interview participants were drawn from the same participant pool. The interview guide was developed by the research team and informed by the CFIR 2.0 domains related to implementation readiness and explored several domains relevant to implementation, including individual characteristics, organizational fit, clinic capacity and resources, implementation supports, adaptability of MBC to the local context, and overall readiness for implementation (10).

Interviews lasted approximately one hour, and all interviews were audio-recorded using the Zoom application with the participant's permission. Three interviews were conducted on Zoom and ten were conducted in-person based on the CDTP staff availability. One researcher led the interview while the second researcher wrote additional notes. Following data collection, interviews were transcribed verbatim in Microsoft Word and de-identified prior to analysis. The same two researchers independently coded transcripts and discussed discrepancies until consensus was reached.

### Qualitative analysis

Interview transcripts were analyzed using the CFIR 2.0, a widely used framework that helps identify factors that can influence implementation success (10). Using a deductive approach, interview data were coded according to the predefined CFIR 2.0 domains and constructs. Coding was conducted manually in Microsoft Word. Two researchers divided the interview transcripts and independently coded their assigned transcripts then each researcher subsequently reviewed the other researcher's coding and provided suggestions or raised differences in interpretation. A third researcher also reviewed all the codes and associated quotes. The researchers discussed these suggestions and resolved differences through discussion and consensus.

Data were coded into the five major CFIR 2.0 domains: Individuals, Innovation, Inner Setting, Outer Setting, Implementation Process. Within these five domains, relevant CFIR 2.0 subdomains were used including characteristics of individuals involved, implementation facilitators, implementation team members, innovation recipients, relative advantage, adaptability, complexity, innovation design, compatibility, culture, work infrastructure, available resources, access to knowledge and information, partnerships and connections, local attitudes, assessing needs, assessing context, teaming, and adapting.

Within each domain and subdomain factors viewed as supporting implementation were classified as facilitators, while factors viewed as hindering implementation were classified as barriers. Findings were organized using the CFIR 2.0 framework to assess implementation readiness and identify organizational barriers and facilitators that could influence successful MBC implementation (Table 2).

**Table 2:** Themes identified in the implementation of MBC guided by CFIR 2.0 domains.

CFIR 2.0 Domains & Definition		Description	Supporting Quotes
Main domain	Sub domain		
1. Individuals: The Roles and Characteristics of Individuals	a. Project Roles	Role at the clinic, knowledge, beliefs (value), confidence, attitudes	“I think oftentimes because we see such a broad range of different cases, and different presentations within a child, such a range of different psychopathology, but also different family system issues across a range of ages, across time and development, where so much is changing so much of the time, I think that we feel as a program that we're very effective and doing really good work. I think historically it has been very hard to quantify it.” - Participant H
	b. Implementation Facilitators	Individuals with subject matter expertise who assist, coach, or support implementation	“ I think it's the professionalism of the staff. You know, we're pretty experienced and qualified. We have the space to see them in person. And we have the kids here during the day” -Participant A
	c. Implementation Team Members	Individuals who collaborate with and support the Implementation Leads to implement the innovation, ideally including Innovation Deliverers and Recipients	“It's an added task. I don't think it's too time-consuming, um, and it'll be kind of divvied up between various team members at various points in time.” - Participant K
	d. Innovation Recipients	Individuals who are directly or indirectly receiving the innovation (patients, family members, caregivers)	“I think from what we've talked about, it will kind of fit naturally into that flow, having them do it upon, first kind of meeting with the family counselor for parents, and then having them kids do it, when they come for their intake. I think those seem like natural things to engage in some, like, measurement.” -Participant G
2. Innovation: Implementation of MBC as the innovation: MBC better than current practices, can be modified to fit the needs of the clinic, complexity of MBC.	a. Innovation Relative Advantage	The innovation is better than other available innovations or current practice.	“Measuring results and efficacy of interventions, there is even a chance to change course or intensify, or otherwise change how the work is being done.” -Participant M
	b. Innovation Adaptability	The innovation can be modified, tailored, or refined to fit local context or needs.	“We have different staff, like myself, doing the intake then than other things. So, there are things already put in place.” Participant A
	c. Innovation Complexity	The innovation is complicated, which may be reflected by its scope and/or the nature and number of connections and steps.	“There's a lot of things that we're asking of our team right now that sometimes do get missed, small, kind of things that might get, that are already being missed and are not consistent.” - Participant K
	d. Innovation Design	Innovation design that is essential for successful implementation	“I think just clear communication, what's happening, and guidelines, and when, and what's expected.” - Participant H
3.Inner setting: CDTP	a.Compatibility	Fit within workflow and structure	“I feel like we’re in a really good position to slide in a couple extra measures.”- Participant L
	b.Culture	Norms, values, team dynamics	“Yeah. I think in general, people are pretty open to change.” - Participant E “We as a program have a recent track record of going through a lot of a lot of changes implementation of a lot of requirements that were that came down to us from a higher level.” - Participant M
	c. Work Infrastructure	Organization of tasks and responsibilities within and between individuals and teams, and general staffing levels, support functional performance of the Inner Setting	“If anything comes up, and then if someone doesn't feel comfortable talking in a larger group, best practice support, and so they have time with [CDTP Clinical supervisor] to talk about anything that's coming up for them.”- Participant J
	d. Available Resources	Staff, technological support (tablets, and support to use it (access to results, Connect Care, RedCAP, PowerBI etc.)	“I think the means are there, the people, I'm sure people can, fit it into their kind of workflow. Time is always more of an issue, but I think the time can be made. I think, from what I understand, it's pretty brief, the measures that we're doing, so I don't see it as being, a huge barrier.”- Participant G
	e. Access to Knowledge and Information	Needs for guidance and/or training to implement and deliver the innovation.	“It's helpful to have some sort of visuals, concrete, real-life examples. And just to speak like, honestly and freely about what's it gonna look like? What do you expect? What are the timeframes? Just as concrete as possible? And then just some explanation of how this is going to help, how this is going to. You know,those kinds of things would be helpful. “- Participant D
4. Outer Setting: System in which program exists (MBC in Recovery Alberta). External influences: policies, patient needs, external pressures.	a. Partnerships & Connections	The Inner Setting is networked with external entities, including referral networks, academic affiliations, and professional organization networks (CBE Dr. Gordon Townsend School, Coast Kids, community outreach programs)	“Do you anticipate that this, the results of this research will impact, even, you know, political decisions around programs that need to be shifted to meet the needs of the population.”- Participant K
	b. Local Attitudes	Sociocultural values (e.g., shared responsibility in helping recipients) and beliefs (e.g., convictions about the worthiness of recipients) encourage the Outer Setting to support implementation and/or delivery of the innovation	“Our priorities are patient care and to deliver excellence in our care.”- Participant L
5. Implementation Process (planning): The activities and strategies used to implement the innovation	a. Assessing Needs	Collect information about priorities, preferences, and needs of team members	“Just so that we know that our information is going somewhere and not just kind of it just communicates that it's valuable.”- Participant K
	b. Assessing Context	Collect information to identify and appraise barriers and facilitators to implementation and delivery of the innovation	“When I'm doing it [measures], I'm having to email a parent, talk to them about it, it just feels like there's a lot of steps to get them to do it, and then when I've gotten it back, it hasn't actually been super valid “- Participant F
	c. Teaming	Strong team collaboration through meetings	“We have our, like, monthly practice support meetings… and then also there's, like, the quality improvement committee… or are, like, community meetings every Wednesday.”- Participant G
	d. Adapting	Modifying the innovation and/or the Inner Setting for optimal fit and integration into work processes.	“I don't have any suggestions. For consistency, it might be worth having the same person [administer measures].” - Participant M

Identified barriers from the CFIR 2.0 analysis were subsequently mapped to the COM-B framework (11) to identify behavioural determinants related to capability, opportunity, and motivation and inform strategies for the research team to address these barriers (Table 3).

**Table 3:** COM-B determinants, barriers and implementation strategies identified during the pre-implementation assessment.

COM-B	Determinant	Barriers	Strategies
Capability	Psychological Capability	Staff identified a need for training on MBC workflow, interpretation of measures, and how to explain MBC to patients and families.	Support sessions by clinic team and MBC training provided by research team Development of educational videos with patient partners on measurement-based care and its importance
Opportunity	Physical Opportunity	Unclear how data will be visualized	Power BI testing using simulated data to assess data visualization and identify issues before implementation.
		Potential technology failures (e.g., Wi-Fi, REDCap, Power BI).	Wi-Fi/REDCap/Power BI contingency workflow- use of paper copies of the measures, and manual entering of data once RedCap available
Opportunity	Social Opportunity	Workflow Concern (Roles and Responsibilities)	Revised staff responsibilities to align measure administration with existing roles and workflow.
Motivation	Reflective Motivation	Uncertainty about whether MBC should be a high priority and whether standardized measures provide additional information beyond clinical judgment.	Discuss the clinical value of MBC with staff, including how standardized measures can complement clinical judgment and support monitoring of patient progress
Motivation	Automatic Motivation	Clinicians forgetting to administer the measures	Automated REDCap email reminders to prompt parents/caregivers to take measures at required time points.

## Results

### Survey results

Thirteen staff members were invited to complete the survey, and ten completed it resulting in a 77% response rate. Most participants identified as women (80%). Clinicians that participated in the survey included the manager, family counsellors, clinical nurses, and psychologists. There were mixed educational degrees (undergraduate degrees- 40%, master's- 40%, and doctorates-20%). The age and years in their profession varied; however, specific age ranges and years in the profession will not be reported to protect participant confidentiality given the small sample size. The survey results are summarized in Table 1.

### Current MBC training and practice

Clinic Staff had mixed responses regarding being trained in the use of MBC and how to use scores for clinical decisions and having sufficient knowledge to be able to interpret MBC score. Most staff (70%) also did not use MBC in their current practice.

### Perceived value and benefits of MBC

Most staff (80%) agreed that MBC was an important and valuable part of treatment and discussing MBC scores with patients and caregivers was important for care and to strengthen the relationship between healthcare providers and patients. The majority of the participants did not perceive any concerns with providing negative feedback to patients and caregivers.

Additionally, most clinic staff trusted the validity and utility of using standardized measures for MBC, however there were mixed responses on whether standardized measures help gather objective information about whether treatment is working.

### Clinic readiness for MBC implementation

Most clinic staff (70%) did agree that MBC aligns with the values of the clinic. However, there were mixed responses on how MBC would align with the clinic workflow, as well as whether MBC is considered a high priority for the clinic. Questions on the processes on MBC implementation such as whether appropriate technology was available, communication regarding the roles of individuals, and sufficient training were answered with mixed responses.

### Qualitative findings

From the readiness interviews, clinicians spoke about their perception of MBC, and how it would work in their program. Table 2 summarizes the themes identified within each CFIR 2.0 domain and subdomain, highlighting the barriers and facilitators that influenced implementation readiness for MBC.

Overall, participants expressed positive attitudes toward the implementation of MBC and perceived it as consistent with the CDTP clinics commitment to evidence-based practice. Survey findings showed that staff generally saw value in monitoring and feedback and in using standardized outcome measures, but there was more uncertainty around the practical aspects of implementation, including technology, training, workload, and role clarity. The interviews helped explain these concerns in more detail as participants described strong multidisciplinary collaboration, leadership support, and established communication structures as important facilitators, while also raising concerns about how MBC would fit into their existing workflow. Participants also felt that MBC could improve on current practices by providing objective information to support clinical decision-making and monitor treatment effectiveness. This contrast was particularly clear in the survey findings: while 70% of respondents agreed that MBC fit with the clinic's values, no respondents agreed that roles were well defined or that adequate technology was available to support implementation.

Findings are organized below according to the five CFIR 2.0 domains: Individuals, Innovation Inner setting, Outer Setting and Implementation Process, were used to guide the analysis.

### Individuals

#### Barriers

Some participants acknowledged that implementation would require staff to adapt to new responsibilities and workflows. Given existing workload demands, participants recognized that it may take time for staff to adjust to new processes.

#### Facilitators

Most participants demonstrated positive attitudes toward MBC and viewed it as consistent with the program's commitment to evidence-based practice. Staff believed MBC would help measure treatment effectiveness, monitor patient progress, and support clinical decision-making. Participants also identified change champions and multidisciplinary team members as important sources of support throughout implementation.

### Innovation

#### Barriers

Some participants anticipated challenges related to integrating MBC into routine practice. Concerns focused on establishing new routines and ensuring that roles and responsibilities were clearly defined among team members.

#### Facilitators

Some participants viewed MBC as an improvement over existing practices because it would provide a structured way to monitor outcomes and evaluate treatment effectiveness. Staff felt that MBC could be incorporated into existing clinic workflows and identified several current processes that could support implementation.

### Inner setting

#### Barriers

Communication challenges across team members and competing workload demands were identified as potential barriers. Participants also expressed a need for additional training and information regarding MBC processes, including how data would be collected, accessed, interpreted, and used in clinical care.

#### Facilitators

Participants described CDTP as having a culture that supports evidence-based practice and is generally receptive to change. Existing staff collaboration, leadership support, practice support meetings, and staffing resources were viewed as strengths that would support implementation. Participants also noted that most staff were already familiar with administering standardized measures throughout the program, making the transition to MBC feel like a more natural extension of existing practice.

### Outer setting

#### Barriers

Participants reported limited awareness of external partnerships or system-level factors that might influence implementation.

#### Facilitators

Participants identified strong alignment between MBC and the broader goal of improving care for children, youth, and families. Participants also felt that MBC could provide valuable information about patient outcomes and help the clinic evaluate the effectiveness of its services.

### Implementation process

#### Barriers

Participants felt that continued support would be important to keep implementation on track over time. They also described potential difficulties administering and collecting measures, particularly when caregivers were involved, highlighting the value of regular check-ins, ongoing support, and opportunities to work through challenges during implementation.

#### Facilitators

Participants identified existing team meetings, practice support sessions, and collaboration with the research team as important supports for implementation. These structures provided opportunities to discuss challenges and share feedback. Participants also felt that workflows could be refined over time to better fit the needs of the clinic.

### Strategies to address barriers

COM-B was used to map identified barriers to capability, opportunity, and motivation and to guide the development of implementation strategies to address these barriers (11).

Findings from the pre-implementation survey readiness assessments with the CDTP helped shape several aspects of implementation planning. One area that required considerable attention was the technology required to support MBC. In collaboration with CDTP staff and technical support teams (Power BI), the research team tested REDCap survey administration on clinic iPads, worked through issues related to software updates and application access, and ensured that REDCap displayed survey data accurately. Before implementation began, simulated data were entered into the system by the research team over a one-week period, three times, to test the workflow from survey completion to data visualization.

Integrating MBC into existing clinic workflows was another important consideration. Early discussions suggested that nurses would be responsible for administering measures; however, further conversations with CDTP staff led to adjustments that better aligned with existing roles and responsibilities. As a result, youth measures were assigned to the Psychometrist and caregiver measures to the Family Liaison, allowing MBC to fit better within current clinical processes.

Participants of the readiness interviews also raised concerns about what would happen if technical issues occurred during implementation, such as internet outages or disruptions to REDCap or Power BI. In response, the research team worked with clinic staff to develop a workflow outlining procedures for different technical scenarios including what to do if REDCap, WIFI or Power BI were down. As a result, paper copies of consent forms and measures were assembled into clinic binders so that data collection could continue if electronic systems were unavailable.

To reduce the burden on staff and caregivers, automated REDCap reminders were created to notify staff when measures were due for administration and caregivers for completion. These reminders were intended to support correct timing for administration and make it easier to incorporate MBC into routine care.

Finally, participants emphasized the importance of feeling confident when introducing MBC to patients and caregivers who may not understand what it is. In response, the research team provided training sessions to help clinic staff understand the principles of MBC, interpret the PROMs, and discuss their purpose with patients and families. In addition, materials were created, including information leaflets, that clinic staff could hand directly to parents and patients. An educational video introducing MBC was also developed for patients and caregivers, with feedback from patient partners. The video was reviewed by clinic staff during the readiness assessment, and their feedback was used to refine its content before implementation and the video will be shown to patients and caregivers before they complete the measures.

## Discussion

This study examined the pre-implementation phase of MBC at the CDTP clinic. Overall, staff viewed MBC positively and felt that it aligned with CDTP emphasis on evidence-based care and continuous improvement. At the same time, participants identified practical barriers related to workflow, workload, roles and responsibilities, training, communication, and technology. Together, these findings suggest that readiness for MBC involves more than just staff support but also how MBC can successfully be integrated into this specific clinics context with roles, processes and resources in mind.

The participants’ generally positive views of MBC are consistent with previous research showing that clinician perceptions and buy-in can play an important role in implementing routine outcome monitoring (13). Staff at CDTP described MBC as useful for monitoring patient progress and supporting clinical decision-making, which is also consistent with previous research describing the value of MBC in providing timely information to guide care (3). Importantly, some interviewees described MBC as complementary to, rather than a replacement for, clinical judgment. This may have contributed to the positive attitudes toward implementation and suggests that presenting MBC as a tool to complement clinical judgment, rather than replace it, may be important when introducing MBC into practice.

Despite this support, some staff identified practical challenges to integrating MBC into routine care. They emphasized that MBC should “fit naturally into that flow” and be “folded into the program” rather than introduced as an additional task. Similar concerns regarding workload, compatibility with existing workflows, and time demands have been identified in previous research on digital MBC in youth and young adult mental health services (14). An important contribution of this work was demonstrating how a structured pre-implementation assessment can be used to translate identified barriers into concrete, context-specific actions before implementation begins. Together, this work suggests that addressing workflow concerns and tailoring implementation strategies to the needs of each clinic may be important for successfully integrating PROMs into routine care and realizing the potential of MBC to improve care delivery. The importance of improving these contextual factors may extend beyond initial implementation to longer-term sustainability of MBC. Jack et al. (15) recently highlighted the limited evidence related to sustaining MBC within CAMHS and described a study examining the contextual conditions and process that could support the sustainment across community CAMHS clinics. The findings from this study may further our understanding of how MBC can be sustained within routine clinical practice and build from the preimplantation findings from this study.

Our approach was informed by the pre-implementation work conducted at The Summit by McCabe et al. (8), but the framework was adapted to reflect the different clinical setting and patient population at CDTP. The readiness assessment and staff survey provided a starting point for examining implementation readiness, while interviews with CDTP staff helped the research team identify which aspects of the approach would fit best within the clinic's existing context such as workflow, staff responsibilities, staff attitudes and available resources. This allowed the pre-implementation process to be guided by an established framework while remaining responsive to the needs, workflows, and perspectives of CDTP staff.

An important contribution of the CDTP readiness assessment was to directly inform implementation planning. By COM-B mapping of identified barriers we found behavioural determinants and guided implementation strategies addressing capability, opportunity, and motivation (11). Strategies included clarifying staff responsibilities, providing MBC training and support, testing data visualization systems, developing alternative workflows for technology failures, and introducing automated reminders for measure administration. This approach aligns with implementation science recommendations to tailor implementation strategies to the barriers within the local context (16, 17). Importantly, this process also incorporated principles of co-design by using staff perspectives and feedback to shape how MBC would be implemented within the CDTP context. For example, rather than implementing a fixed MBC workflow at the clinic, staff feedback led to changes in who would administer the measures, how technical problems would be managed, and what training and supports should be provided. This allowed the implementation approach to address barriers identified specifically at CDTP rather than relying on strategies developed for other settings. This approach is consistent with previous research examining barriers and facilitators to the implementation of MBC in youth mental health settings (18), which highlights the importance of understanding the organizational factors that can influence the use of MBC with youth. Rather than viewing readiness only in terms of staff acceptance, the assessment provided a structured approach to identify specific changes and supports needed to facilitate implementation within the CDTP clinic.

### Person-centered care

The pre-implementation phase and broader INSIGHT project also reflect a person-centred and co-design approach, consistent with the focus of the Person-Centred Care team at the University of Calgary. Person-centred care emphasizes putting the needs and perspectives of individuals at the centre of care and research (19). In this phase, this meant taking the experiences and perspectives of individuals involved in implementing MBC into account and tailoring the integration of MBC into their unique clinical setting.

Overall, the findings from pre-implementation highlight the importance of understanding the individual clinical setting before introducing MBC in child and adolescent mental health care. Rather than assuming that positive attitudes toward MBC would translate into successful implementation, the assessment helped identify specific contextual and behavioural factors that could affect integration of MBC into routine practice. Incorporating staff perspectives through a co-design approach provided an opportunity to identify implementation strategies that were responsive to the needs of this specific clinical setting and the individuals involved in delivering MBC.

### Strengths

A key strength of this study was the use of a mixed-methods readiness assessment that combined an anonymous staff survey with individual interviews to provide an understanding of implementation readiness. The sequential approach allowed findings from the staff survey to inform the interview questions, providing an opportunity to explore survey responses in greater depth. Conducting the assessment before implementation allowed barriers and facilitators to be identified and addressed.

Another strength was the high level of engagement from the clinical team throughout the readiness assessment. Engaging clinicians throughout the planning process can help improve the acceptability and feasibility of implementation efforts by ensuring that implementation strategies reflect the experiences and priorities of those who will ultimately deliver it (20, 21). Perspectives were gathered from clinicians representing a range of roles, including Clinical Supervisor, Family Liaison, Administrative Support, Nurses, Psychologists and Psychometrists, as well as clinic leadership, providing a good understanding of implementation readiness across the clinic. This collaborative approach ensured that the implementation plan reflected the experiences and priorities of the staff who would ultimately be responsible for delivering MBC in routine practice.

The readiness assessment was grounded in the CFIR 2.0 framework, providing a structured approach to identifying implementation determinants (10). Rather than acting solely as an exploratory exercise, the findings were immediately translated into practice. A further strength was the direct translation of identified barriers and facilitators into concrete implementation strategies. CFIR findings were immediately put into practice in collaboration with clinic staff. This demonstrates the practical value of readiness assessments in supporting implementation strategies that are adapted to the local context and may improve the feasibility and sustainability of MBC.

### Limitations

This study has limitations. First, the small sample size of 13 staff participants from a single clinic limits the generalizability and transferability of the findings to other clinical settings. Although the study was intentionally focused on understanding implementation readiness within one clinic, the contextual nature of these findings should be considered when applying them to other settings, where workflows, resources, and organizational contexts may differ. Second, demographic characteristics such as age ranges and years in the profession were not reported to protect participant confidentiality given the small sample size. While this approach was necessary to maintain confidentiality, the limited demographic information restricts the ability to fully characterize the sample and consider how participant characteristics may have influenced the findings. Finally, some survey items had missing responses (*n* = 8 or *n* = 9). Although missing responses may limit the interpretation of individual survey items, the research team reviewed the anonymous survey results prior to conducting the staff interviews. These findings provided contextual information that helped inform the interviews and supported further exploration of topics identified in the survey. Nevertheless, the presence of missing data should be considered when interpreting the survey findings.

## Conclusion

This pre-implementation assessment identified key barriers and facilitators to implementing MBC within CDTP and demonstrated the value of understanding clinic facilitators and barriers towards MBC implementation. Findings were consistent with previous research highlighting the importance of clinician engagement, workflow integration, and organizational and technological support for routine outcome monitoring in child and adolescent mental health settings (8, 22). The identified barriers helped guide the development of implementation strategies, demonstrating how pre-implementation assessments can be used to identify clinic-specific challenges and develop strategies that address them. Future stages of this project will provide an opportunity to assess how well the pre-implementation assessment prepared CDTP for MBC implementation and how well the planned strategies fit within the clinic's existing context and workflows. These findings will provide a clearer understanding of which strategies supported implementation and which factors continued to hinder it. In turn, these findings may provide a starting point for structuring the initial stages of MBC implementation, while allowing individual programs to adapt the approach to their own way.

## Data Availability

The raw qualitative data generated and analyzed during this study are not publicly available due to the potential for participant identification and confidentiality requirements. Anonymized data is available upon request.
